# Comparative Chloroplast Genome Analyses of the Winter-Blooming Eastern Asian Endemic Genus *Chimonanthus* (Calycanthaceae) With Implications For Its Phylogeny and Diversification

**DOI:** 10.3389/fgene.2021.709996

**Published:** 2021-11-30

**Authors:** Abbas Jamal, Jun Wen, Zhi-Yao Ma, Ibrar Ahmed, Abdullah, Long-Qing Chen, Ze-Long Nie, Xiu-Qun Liu

**Affiliations:** ^1^ Key Laboratory of Horticultural Plant Biology (Ministry of Education), College of Horticulture and Forestry Science, Huazhong Agricultural University, Wuhan, China; ^2^ Department of Botany, National Museum of Natural History, MRC166, Smithsonian Institution, Washington, DC, United States; ^3^ Alpha Genomics Private Limited, Islamabad, Pakistan; ^4^ Department of Biochemistry, Faculty of Biological Sciences, Quaid-i-Azam University, Islamabad, Pakistan; ^5^ Southwest Engineering Technology and Research Center of Landscape Architecture, State Forestry Administration, Southwest Forestry University, Kunming, China; ^6^ Key Laboratory of Plant Resources Conservation and Utilization, College of Biology and Environmental Sciences, Jishou University, Jishou, China

**Keywords:** chloroplast genomics, correlations, phylogeny, incipient radiation, divergence time, subtropical forest biome assembly

## Abstract

*Chimonanthus* of Calycanthaceae is a small endemic genus in China, with unusual winter-blooming sweet flowers widely cultivated for ornamentals and medicinal uses. The evolution of *Chimonanthus* plastomes and its phylogenetic relationships remain unresolved due to limited availability of genetic resources. Here, we report fully assembled and annotated chloroplast genomes of five *Chimonanthus* species. The chloroplast genomes of the genus (size range 153,010 – 153,299 bp) reveal high similarities in gene content, gene order, GC content, codon usage, amino acid frequency, simple sequence repeats, oligonucleotide repeats, synonymous and non-synonymous substitutions, and transition and transversion substitutions. Signatures of positive selection are detected in *atpF* and *rpoB* genes in *C. campanulatus*. The correlations among substitutions, InDels, and oligonucleotide repeats reveal weak to strong correlations in distantly related species at the intergeneric levels, and very weak to weak correlations among closely related *Chimonanthus* species. Chloroplast genomes are used to reconstruct a well-resolved phylogenetic tree, which supports the monophyly of *Chimonanthus*. Within *Chimonanthus*, *C. praecox* and *C. campanulatus* form one clade, while *C. grammatus*, *C. salicifolius*, *C. zhejiangensis*, and *C. nitens* constitute another clade. *Chimonanthus nitens* appears paraphyletic and is closely related to *C. salicifolius* and *C. zhejiangensis*, suggesting the need to reevaluate the species delimitation of *C. nitens*. *Chimonanthus* and *Calycanthus* diverged in mid-Oligocene; the radiation of extant *Chimonanthus* species was dated to the mid-Miocene, while *C. grammatus* diverged from other *Chimonanthus* species in the late Miocene. *C. salicifolius*, *C. nitens* *(a)*, and *C. zhejiangensis* are inferred to have diverged in the Pleistocene of the Quaternary period, suggesting recent speciation of a relict lineage in the subtropical forest regions in eastern China. This study provides important insights into the chloroplast genome features and evolutionary history of *Chimonanthus* and family Calycanthaceae.

## Introduction

Due to its uniparental inheritance, moderate evolutionary rate, and highly conserved genome structure and gene content in most land plants, the chloroplast genome has been widely used in phylogenetic studies (Jansen et al., 2007; Daniell et al., 2016; Valcárcel and Wen, 2019; Wang Y. B. et al., 2020; Mehmood et al., 2020c), DNA barcoding (Ahmed et al., 2013; Bi et al., 2018; Abdullah et al., 2020e), dating divergence times (Zhang et al., 2019; Ahmed et al., 2020; Mehmood et al., 2020c), and studying the origins of horticulturally important plants, e.g., *Chrysanthemum* cultivars (Ma et al., 2020). With the rapid advancements in next-generation sequencing, the availability of cp genome sequences has dramatically increased for flowering plants, offering opportunities for comparative evolutionary and systematic studies, and improvement of horticultural plant breeding (Sonah et al., 2011; Ahmed, 2015; Xiong et al., 2015; Wen et al., 2018; Thode et al., 2020).

The Calycanthaceae (sweetshrubs or spicebushes) are a small family of flowering plants, which is sister to the remaining six families of the order Laurales of the Magnoliids (Renner, 1999; Chase et al., 2016). The family contains 10 known species in three genera (Zhou et al., 2006; Christenhusz and Byng, 2016). *Calycanthus* comprises three species, *Cal. floridus* L. and *Cal. occidentalis* Hook. and Arn. in North America and *Cal. chinensis* W.C. Cheng and S.Y. Chang in eastern China. The latter species was sometimes recognized as a member of the monotypic genus *Sinocalycanthus*, based on floral morphology (Cheng and Chang, 1964). However, the chloroplast DNA analyses supported *Sinocalycanthus* as part of *Calycanthus* (Wen et al., 1996; Wen, 1999). The Chinese endemic genus *Chimonanthus* consists of six species: *C. praecox* L., *C. campanulatus* R.H. Chang and C.S., *C. nitens* Oliv., *C. salicifolius* S.Y. Hu., *C. grammatus* M.C. Liu, and *C. zhejiangensis* M.C. Liu. The phylogenetic relationships among the latter four species remain poorly resolved [see (Zhou et al., 2006) for details]. *Chimonanthus* species are widely cultivated as ornamental plants with high economic value, and several species of the genus are also utilized for their medicinal properties (Sun et al., 2017; Zhang X. et al., 2017, Zhang et al., 2017 S.). *Idiospermum* is a highly distinctive monotypic genus from Queensland, Australia, consisting of *Idiospermum australi*ense (Diels) S.T. Blake (Blake, 1972; Carlquist, 1983). Based on DNA markers and morphological characters, *Idiospermum* is sister to the clade of *Chimonanthus* and *Calycanthus* (Li and Li, 2000; Li et al., 2004; Zhou et al., 2006).

The previous molecular phylogenetic studies have resolved *C. praecox* and *C. campanulatus* as a clade, while phylogenetic relationships among the rest of *Chimonanthus* species remain poorly resolved (Zhou et al., 2006), partly due to lack of informative characters for all species. In the context of inferring the temporal diversification of Calycanthaceae, Zhou et al. (2006) only used a few markers with inconsistent age estimates. Therefore, it is important to use a large region of the chloroplast genomes to provide more precise age estimates (Nikiforova et al., 2013; Middleton et al., 2014; Wen et al., 2018; Zhang et al., 2019). So far, only two chloroplast genomes of *Chimonanthus* have been reported (Dong et al., 2020).

We herein newly report fully assembled and annotated chloroplast genomes of four species: *C. campanulatus*, *C. grammatus*, *C. salicifolius*, and *C. zhejiangensis*. We have also assembled the chloroplast genome of one additional accession of *C. nitens* collected from Jiangxi, China, and included two previously published cp genomes in the analyses. The objectives of our study are to characterize and perform comparative analyses of the chloroplast genomes, evaluate correlations among mutational events, infer the phylogenetic relationships within *Chimonanthus* with all six recognized species of the genus sampled, and estimate their divergence times to explore the evolutionary diversification of the endemic genus from China. Our assemblies and data will serve as useful genetic resources for future research in this horticulturally unique and systematically important genus *Chimonanthus* and the family Calycanthaceae of the Magnoliids.

## Materials and Methods

### Taxon Sampling and Sequencing

Fresh and healthy leaves of *C. grammatus*, *C. salicifolius*, and *C. nitens* were collected from natural populations located at Anyuan (25.28°N, 115.45°E), Yushan (28.77°N, 118.12°E), and Guixi (27.90°N, 117.20°E) in Jiangxi Province, China, respectively. *C. campanulatus* leaves were collected from the Ornamental Horticulture Nursery, Huazhong Agricultural University, Wuhan (30.47°N, 114.36°E), China, while those of *C. zhejiangensis* were sampled from its native habitat at Longquan (28.13°N, 119.03°E) in Zhejiang Province, China (Supplementary Table S1). The voucher specimens were deposited at the Herbarium of the Key Laboratory of Horticultural Plant Biology (Ministry of Education), College of Horticulture and Forestry Science, Huazhong Agricultural University (China). No specific permissions were required for all the samples, which are neither protected nor privately owned, and the field study did not involve protected or endangered species.

Total genomic DNA was isolated from fresh or silica gel-dried leaves of the five *Chimonanthus* species (including one accession of *C. nitens*) using the CTAB method (Doyle and Doyle, 1987). Electrophoresis with 1% agarose gel was used to evaluate the DNA quality. DNA samples were sent to Novogene (Beijing, China) for whole-genome sequencing on Illumina HiSeq platform, with the PE 150 run.

### Genome Assembly and Annotation

The quality of the generated short read data was assessed using FastQC (Andrews et al., 2015) with default parameters. *Chimonanthus praecox* cp genome (NCBI accession number MH377057) was used for initial mapping of the short reads using the Burrows–Wheeler Alignment (BWA) algorithm (Li and Durbin, 2009). The alignment file (SAM format) was visualized in Tablet (Milne et al., 2009) to check for presence of short reads coming from the chloroplast genomes and to obtain an estimate of average coverage depth, a statistical measure needed for the downstream assembly process. The genomes were assembled following the methodology reported earlier (Ahmed et al., 2012; Iram et al., 2019; Abdullah et al., 2020b). Briefly, the chloroplast genomes were assembled using the Velvet (v.1.2.10) tool (Zerbino and Birney, 2008) to form contigs. Assembly process was reiterated thrice for each individual samples by selecting different kmer sizes, including 71, 91, and 111 kmer lengths, and selecting coverage cutoff option of 100 during the Velvet assembly process. The assembled contigs were imported in Geneious 8.1 (Kearse et al., 2012) and further assembled from large scaffolds into full-length genomes. The boundaries of large single copy (LSC), small single copy (SSC), and the two inverted repeats (IRa and IRb) were visually curated. The assembled genomes were curated by mapping the original short reads to their respective assembled genomes using BWA mapping and visualizing in Tablet as given above. Average coverage depth statistics were visually recorded in Tablet for all the genomes. The assembled genomes were annotated using online annotations GeSeq (Tillich et al., 2017), whereas tRNA genes were further verified by tRNAscan-SE v.2.0.3 (Schattner et al., 2005) using default parameters. The annotations of each protein-coding gene were further validated with homologous genes using BLAST of the National Center for Biotechnology Information. Moreover, the annotations of GeSeq were also checked by comparing with previously reported genome of *C. praecox* using the Multiple Alignment Fast Fourier Transform (MAFFT) alignment (Katoh et al., 2005). The assembled chloroplast genomes were circularized using OGDRAW (Greiner et al., 2019). The fully assembled and annotated cp genomes of the five species were submitted to the GenBank database of NCBI and given accession numbers MW166216 through MW166220.

### Comparative Analyses of Chloroplast Genomes

We used Geneious R8.1 (Kearse et al., 2012) to compare genomic features, including sizes of LSC, SSC, and IRs, and determine amino acid frequency and codon usage. The junctions of single copy and inverted repeat regions were also analyzed in Geneious R8.1. We analyzed microsatellite repeats using MISA-web (Beier et al., 2017) with repeat units as follows: mononucleotide repeats ≥ 10, dinucleotide ≥ 5, trinucleotide ≥ 4, tetranucleotide, pentanucleotide, and hexanucleotide ≥ 3 (Mehmood et al., 2020a; Shahzadi et al., 2020). In addition, the oligonucleotide repeats were analyzed using the REPuter (Kurtz et al., 2001) tool by selecting a repeat length of 30 bp and determining all four types of repeats (forward, reverse, complimentary, and palindromic) with minimum 90% identity (Abdullah et al., 2019a).

The contraction and expansion of the IR regions at the junctions of four main parts (LSC/IRb/SSC/IRa) of the cp genomes were inspected and plotted with IRscope (Amiryousefi et al., 2018a). The possibility of large-scale genomic rearrangements among the studied *Chimonanthus* cp genomes was also investigated by generating a multiple sequence alignment of the six chloroplast genomes using MAUVE multiple sequence alignment (Darling et al., 2004) embedded in Geneious R8.1. One IR region (IRa) was excluded in the alignment for this comparison. Large-scale contraction and expansion in inverted repeats at the junctions of inverted repeats with single copies were compared in Geneious 8.1.

We determined transition (Ts), transversion (Tv) substitutions, and their ratio (Ts/Tv) in 78 protein-coding genes. For this purpose, we concatenated the protein-coding genes of all five species. The sequences of the concatenated protein-coding genes of these species were pairwise aligned to *C. praecox* by MAFFT (Katoh et al., 2005) plugin provided in Geneious 8.1 (Kearse et al., 2012). The substitution types were determined from each alignment in Geneious R8.1. We also determined the rate of synonymous (Ks), non-synonymous (Ka) substitutions, and their ratio (Ka/Ks) in 78 protein-coding genes. We extracted and aligned protein-coding genes from all five species. The chloroplast genome of *C. praecox* was used as a reference, and the rates of evolution of protein-coding genes were recorded. The data were interpreted in terms of purifying selection (Ka/Ks < 1), neutral evolution (Ka/Ks = 1), and positive selection (Ka/Ks > 1) (Henriquez et al., 2020b).

### Correlations Among Mutational Events in Calycanthaceae

We determined correlations among substitutions, InDels, and oligonucleotide repeats at the family and genus levels in pairwise comparison using the same approach used previously (Abdullah et al., 2020a). The chloroplast genome of *Idiospermum australiense* was used as reference for family-level comparison. The cp genome of *Calycanthus chinensis* was used as reference in comparison of two species of *Calycanthus*, whereas the cp genome of *Chimonanthus campanulatus* was used as reference for all the species of genus *Chimonanthus*. We also expressed the strength of the correlations as negligible or very weak (0.1–0.19), weak (0.20–0.29), moderate (0.30–0.39), strong (0.4–0.69), very strong (0.70–0.99), and perfect (1.0) following studies by Abdullah et al. (2020d, 2020a).

### Phylogenetic Inference, Bayesian Dating, and Fossil Calibration

A phylogenetic analysis was performed with the maximum-likelihood method (Stamatakis, 2014) using the 10 cp genomes in the family, comprising seven *Chimonanthus* samples representing all six species (two cp genomes for *C. nitens*), two *Calycanthus* species, and one *Idiospermum* species. Chloroplast genomes of *Cal. chinensis*, *Cal. floridus*, *C. praecox*, *C. nitens (a)*, and *Idiospermum australiense* were selected from NCBI (Goremykin et al., 2003; Dong et al., 2020). Full-length cp genomes of all these species were aligned using MAFFT (Katoh et al., 2005) after removal of one IR. We removed indels from the alignment to construct the phylogenetic relationships based only on substitution mutations (Ahmed et al., 2012; Henriquez et al., 2020b). JModelTest2 (Darriba et al., 2012) was used to infer the best fit model on the alignment. A maximum likelihood tree was built with 100 bootstrap replicates, using IQ-tree (Nguyen et al., 2015), and the tree was refined using TreeDyn (Chevenet et al., 2006).

To estimate divergence times for taxa in Calycanthaceae, the maximum likelihood tree obtained from whole chloroplast genomes was used as input to perform the Bayesian dating analyses in the BEAST v1.7.5 package using uncorrelated relaxed clock model (Drummond et al., 2006; Drummond and Rambaut, 2007). Rate variation among sites was modeled using a gamma distribution with four rate categories in the GTR model with a birth–death speciation tree prior and an uncorrelated lognormal distributed relaxed clock model (Drummond et al., 2006). Two independent MCMC runs were performed for 10 million generations each and sampling for every 1,000 generations. Samples from the two chains, which yielded similar results, were combined, and convergence of the chains was checked using the program Tracer version 1.7.1 (Rambaut et al., 2018). The effective sample size (ESS) values of all parameters were greater than 200, indicating a sufficient level of sampling. After the discarding of ca. 15% burn-in, the rest sampled posterior trees were summarized to generate a maximum clade credibility tree using the program TreeAnnotator 1.8.5 (Drummond and Rambaut, 2007) with a PP limit of 0.5 and mean node heights. The means and 95% higher posterior densities (HPD) of age estimates were obtained from the combined outputs using Tracer. FigTree version 1.4.0 (Rambaut, 2012) was used to view the BEAST tree.

The oldest fossil record of Calycanthaceae was reported from the Potomac group in the Early Cretaceous (Early or Middle Albian), based on charcoalified flower *Virginianthus calycanthoides* (Friis et al., 1994). Cladistic analyses by Crepet et al. (2005) suggested that *Virginianthus* may lie on the Laurales stem lineage rather than Calycanthaceae and instead placed a new fossil *Jeresyanthus calycanthoides* described from the Late Cretaceous (∼90 Ma) in New Jersey within Calycanthcaeae. Other fossils belonging to Calycanthaceae, include *Araripia florifera* described from the Early Cretaceous Crato Formation in Brazil (Mohr and Eklund, 2003), and a potential Miocene (∼16 Ma) fossil fruit from Germany (Mai, 1987). *Araripia* may represent an extinct lineage within or close to the Laurales, as it shares features not only with Calycanthaceae but also with several other families of Laurales including *Hernandiaceae* and *Lauraceae* (Mohr and Eklund, 2003). Based on these fossils, the emergence and early diversification of Calycanthaceae might have occurred between 90 and 110 Ma in the Cretaceous.

We used two approaches to calibrate the divergence estimates. First, the minimum age for Calycanthaceae was set to 108 Mya, based on secondary calibration (Dong et al., 2020) with normal prior mean = 108 and sigma = 5.0. This age is also in accordance with the fossil *Araripia florifera* representing Calycanthaceae (Mohr and Eklund, 2003). Second, the minimum age for Calycanthaceae was set to 90 Mya, based on the fossil record of *Jereysanthus calycanthoides* (Crepet et al., 2005) with lognormal mean = 1.0, standard = 1.25, and offset = 90.0.

## Results

### Comparative Analyses of the Chloroplast Genomes

The number of short reads and data produced per sample are summarized in Supplementary Table S2. The table also gives the average coverage depth information of the short reads when mapped to their respective assembled cp genomes. For the paired-end 150 bp run, the total data obtained ranged from 7.1 to 9.5 Gb, and the number of short reads ranged from 19.21 to 25.87 million per sample. The phred quality of the data for all samples remained above 35. Very high average coverage depth (ranging from 1,429× to 6,270×) ensures the accuracy of the assembled genomes.

The sizes of the cp genomes within *Chimonanthus* did not show large variation and ranged from 153,010 bp (*C. campanulatus*) to 153,299 bp (*C. grammatus*). The LSC regions ranged from 86,676 bp (*C. campanulatus*) to 86,928 bp (*C. nitens*); SSC from 19,756 bp (*C. campanulatus*) to 19,795 bp (*C. salicifolius and C. zhejiangensis*); and IRs from 23,275 bp (*C. salicifolius*) to 23, 289 bp (all other species, except *C. praecox*) (Table 1). Notable size differences for the LSC, SSC, and IR regions were observed between cp genomes of the two *C. nitens* accessions (Table 1).

**TABLE 1:** Sizes (bp) of different regions of the assembled chloroplast genomes.

Species	Total size	Large single copy (LSC)	Small single copy (SSC)	Inverted repeat (IR)	NCBI accession number
*C. campanulatus*	153,010	86,676	19,756	23,289	MW166216 (This study)
*C. grammatus*	153,299	86,927	19,794	23,289	MW166217 (This study)
*C. nitens (a)*	153,250	86,882	19,708	23,330	NC_042745 (NCBI)
*C. nitens (b)*	153,298	86,928	19,792	23,289	MW166220 (This study)
*C. praecox*	153,252	86,912	19,766	23,287	MH377057 (NCBI)
*C. salicifolius*	153,243	86,898	19,795	23,275	MW166218 (This study)
*C. zhejiangensis*	153,284	86,911	19,795	23,289	MW166219 (This study)

**FIGURE 1:**
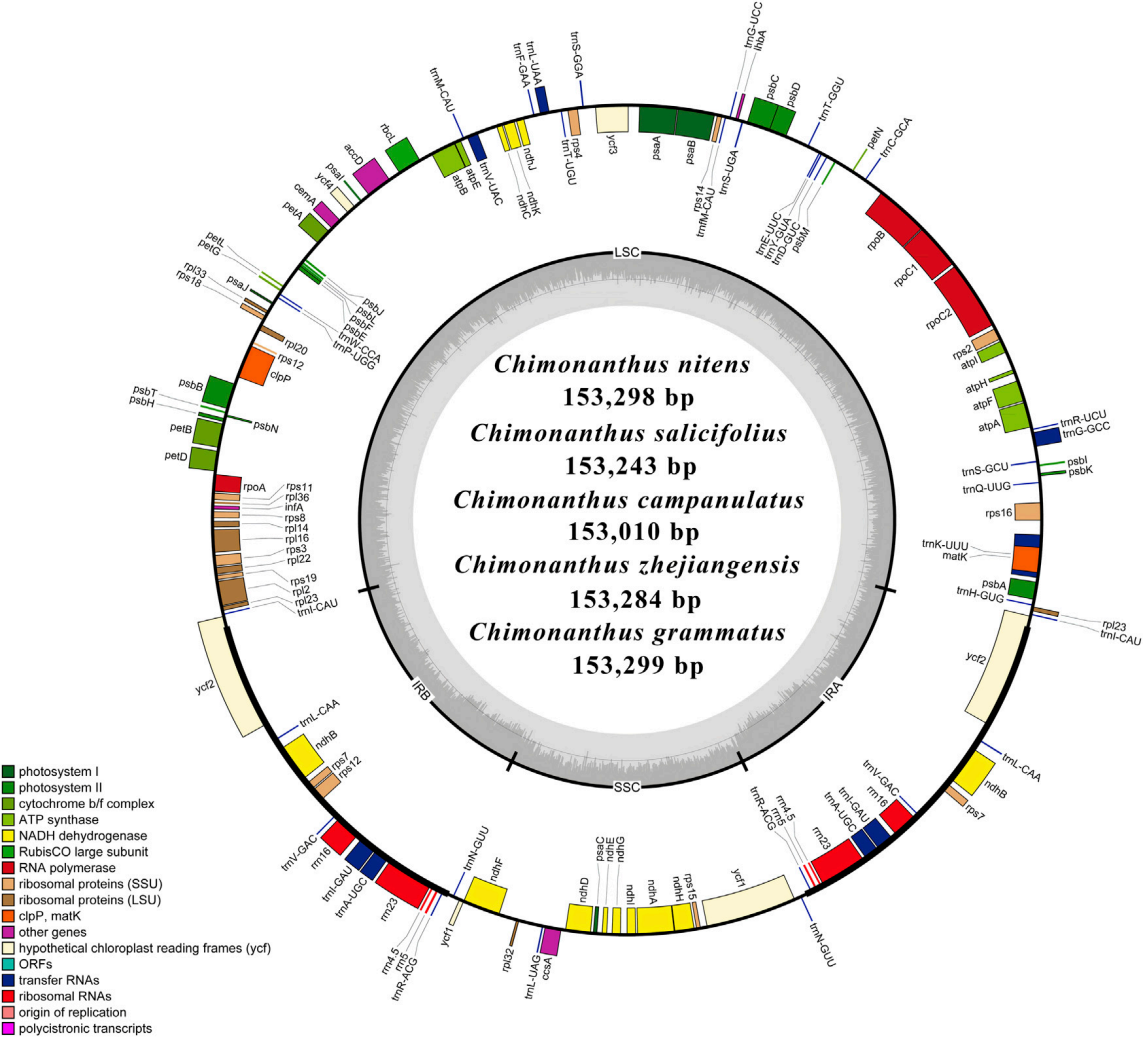
Schematic representation of the *de novo* assembled circular chloroplast genomes of five *Chimonanthus* species. Genes present inside the circle are transcribed counterclockwise, whereas genes present outside the circle are transcribed clockwise. Genes are color-coded based on their role in different pathways, as given in the key. Large single copy (LSC), inverted repeat b (IRb), small single copy (SSC), and inverted repeat a (IRa) of the inner circle represent quadripartite structure of cp genomes. Dark grey area in the inner circle represents average guanine–cytosine (GC) content, while light grey represents AT content.

A representative circular map of the chloroplast genomes of the five *Chimonanthus* species assembled in this study is given in Figure 1. All five species were found to be highly conserved in terms of gene organization, gene content, and intron content. All species exhibited 113 unique genes, including 79 protein-coding, 30 tRNA, and four rRNA genes (Supplementary Table S3). We recorded 15 duplicated genes in the IRs of all genomes. Among these duplicated genes, four were protein coding, seven encoded tRNAs, and four encoded rRNA genes. Hence, the total number of genes in each of the *Chimonanthus* species was 128. We detected two introns in *clpP*, *rps12*, and *ycf3* genes. A complete open reading frame (ORF) of the *infA* gene was present in all species (Supplementary Table S3). The guanine–cytosine (GC) content of the complete chloroplast genomes and of all regions showed high similarities among the species, whereas fluctuation in GC content was observed within the different regions of the same chloroplast genome. The GC content of coding regions, rRNAs, and tRNAs also showed high similarities among the species (Supplementary Table S4).

### Dynamics at the Boundaries of Inverted Repeats and Single Copy Regions

**FIGURE 2:**
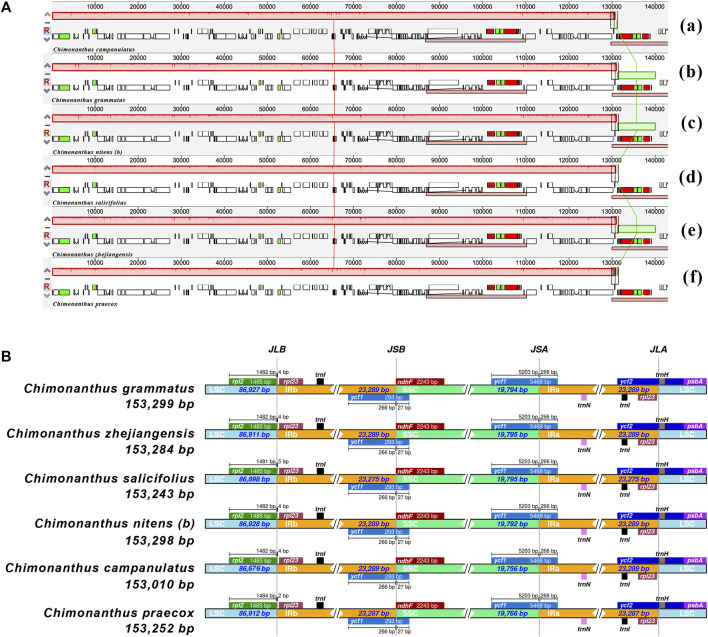
Comparative analysis of gene order and junction sites in *Chimonanthus* chloroplast genomes. **(A)** Mauve alignment of six *Chimonanthus* chloroplast genomes. Within each of the alignments, local collinear blocks are represented by blocks of the same color connected by lines. (a) *C. campanulatus*, (b) *C. grammatus*, (c) *C. nitens (b)*, (d) *C. salicifolius*, (e) *C. zhejiangensis*, and (f) *C. praecox*. **(B)** The IRscope based analyses provide insight into similarities and variations at junction sites of cp genomes. For each species, genes present on the top of their corresponding track transcribed in positive strand from right to left direction whereas genes present below their respective track transcribe on negative strand from left to right direction. JLA, JLB, JSA, and JSB denote corresponding junctions between the LSC, SSC, and IR regions. The genes extending from one region to another region of chloroplast genomes are shown with the T bar above or below the solid lines. The size of T bars corresponds to the length of the genes or gene fragments present in the different regions. The plotted genes and distances in the vicinity of the junction sites are the scaled projection of the genome.

The chloroplast genomes showed overall similarities at all four junctions of the single copy and inverted repeat regions in all six *Chimonanthus* species. No significant differences were observed across all six species at these junctions (Figure 2B). At the JLA (junction of large single copy with IRa), *trnH* gene was found in LSC 11–14 bases downstream at the start of the LSC in different species, while *rpl23* gene was present 27–30 bases prior to the IRa end in different species. At the JLB (LSC–IRb junction), *rpl2* gene started two to four bases inside IRb and extended in LSC. At the JSA (SSC-IRa junction), *ycf1* gene was found to extend from IRa to SSC; the first 266 bases of this gene were found in all the six chloroplast genomes. This resulted in a truncated copy (or a pseudogene) of *ycf1* gene at the JSB (SSC-IRb junction) in IRb, whereas *ndhF* gene was found completely in the SSC region, ending exactly at the junction. This conserved structure was also confirmed using colinear block analyses of Mauve (Figure 2A).

### Analyses of Codon Usage and Amino Acid Frequency

The codon usage analyses revealed high encoding efficacy for those codons that end with A/T at the 3′ end as opposed to codons that end with C/G at the 3′. The amino acid frequency analyses revealed that leucine was the highest (>10%) encoded amino acid followed by iso-leucine (>8%), whereas cysteine (1) and tyrosine (1.5%) were among the least encoded amino acids (Supplementary Table S4).

### Repeats Analyses

The analyses of microsatellites revealed 49–54 repeats in the six cp genomes (Supplementary Table S5). Most of the repeats existed in the non-coding regions of LSC, followed by SSC, and then IR (Supplementary Figure S1A). Mononucleotide A/T repeats were most abundant in all species, especially in *C. campanulatus*, followed by dinucleotide and trinucleotide repeats. Notably, mononucleotide G/C repeats were only found once or twice in the genomes. Most of the microsatellites were in A/T rich regions (Supplementary Table S5). The analyses of oligonucleotide repeats (30 nucleotides or longer in length) showed that most of the repeat types present in the chloroplast genomes were forward repeats, followed by palindromic repeats. Very few reverse repeats were found only in two cp genomes, and complimentary repeats were not found in the genomes (Supplementary Figure S1B).

### Analyses of Substitution Types

We recorded a higher number of transition (*Ts*) than transversion (*Tv*) substitutions. The ratios of *Ts*/*Tv* ranged from 1.2 to 1.55 (Table 2). The majority of *Ts* substitutions were promoted by C/T mutations except in *C. campanulatus* where A/G mutations were slightly higher than C/T mutations. *Tv* substitutions were found to be related to A/C and G/T rather than to A/T and C/G (Table 2). For *K*_*s*_ and *K*_*a*_, we found a higher average of *K*_*s*_ than *K*_*a*_ on 78 protein-coding genes in the *Chimonanthus* species. Only *atpF* and *rpoB* genes showed positive selection (*K*_*a*_/*K*_*s*_ ratio of 1.92 and 1.23, respectively) in *C. campanulatus* chloroplast genome. The *rbcL* gene in *C. grammatus* showed neutral selection (*K*_*a*_/*K*_*s*_ ratio of 0.96), while the *matK* gene in *C. grammatus*, *C. nitens*, and *C. zhejiangensis* showed approximate neutral selection (*K*_*a*_/*K*_*s*_ ratio of 0.86). All other genes in all species showed strong purifying selection (Supplementary Table S6).

**TABLE 2:** Comparison of transition and transversion mutations in *Chimonanthus* species.

Substitution types	*C. zhejiangensis*	*C. salicifolius*	*C. nitens (b)*	*C. grammatus*	*C. campanulatus*
A/C	57	60	57	55	48
C/T	106	110	109	112	72
A/G	101	103	103	102	75
A/T	16	10	15	13	12
C/G	12	13	12	15	13
G/T	58	58	61	55	49
Ts/Tv	1.45	1.51	1.46	1.55	1.20

### Quantitative and Qualitative Analyses of Correlations Among Mutational Events

We performed four comparisons at the family level and six comparisons at the genus level to unravel the quantitative and qualitative correlations among the species of Calycanthaceae.

**FIGURE 3:**
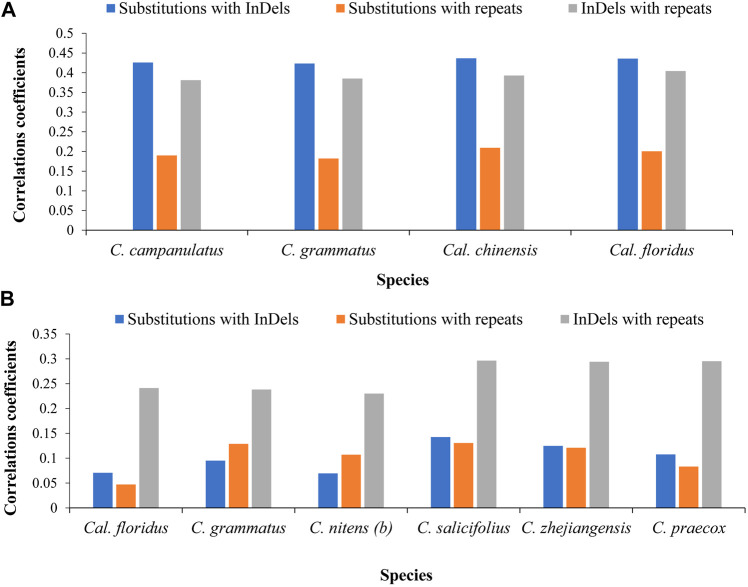
Correlations among mutational events at family and genus levels. **(A)** Correlations among distinctly related species of family level. The chloroplast genome of *Idiospermum australiense* was used as reference for all comparisons. **(B)** Correlations among closely related species at genus level. The *Calycanthus chinensis* was used as reference in comparison of two species of *Calycanthus*, whereas the cp genome of *Chimonanthus campanulatus* was used as reference for all species of *Chimonanthus*. The x-axis shows name of the species, whereas the y-axis shows the value of correlations coefficient.

At the family level, we found strong correlations between substitutions and InDels in all four comparisons with an average of 0.43. The correlations between InDels, and repeats were moderate in two comparisons while near to strong in two comparisons. The average of correlations was found at 0.39. Correlations between substitutions and repeats were very weak in two comparisons, whereas weak correlations were observed in two comparisons. The average of correlations was found at 0.195 (Figure 3A).

At the genus level, we observed negligible/very weak or weak correlations. We recorded negligible correlations between substitutions and InDels (0.07–0.14) and between substitutions and repeats (0.04–0.13) in pairwise comparisons of all species, whereas we recorded weak correlations between InDels and repeats (0.23–0.29). Here, our results revealed weak correlations between closely related species (Figure 3B).

Qualitative analyses revealed that, at the family level, 98.36–98.90% of InDels co-occurred with substitutions in the same bins, whereas 95.30–97.01% of repeats co-occurred with substitutions, and 56.41–58.97% of repeats co-occurred with InDels in the same bins. At the genus level, 53.23–69.57% of InDels coexisted with substitutions, whereas 8.93–17.85% of repeats coexisted with InDels, and 41.96–56.25% of repeats coexisted with substitutions (Table 3). The distributions of substitutions, InDels, and repeats in 250-bp bins are shown in Supplementary Table S7.

**TABLE 3:** The co-occurrence of InDels with substitutions, and of repeats with substitutions and InDels in family Calycanthaceae.

Species	SNPs with InDels (%)	InDels with repeats (%)	SNPs with repeats (%)
Family level			
*Chimonanthus campanulatus*	98.90	56.41	96.15
*Chimonanthus grammatus*	98.36	57.27	95.73
*Calycanthus chinensis*	98.40	58.97	95.30
*Calycanthus floridus*	98.39	58.12	97.01
Genus level			
*Calycanthus floridus*	53.23	21.12	49.57
*Chimonanthus grammatus*	60.38	17.41	54.91
*Chimonanthus nitens (b)*	65.22	8.93	53.13
*Chimonanthus salicifolius*	69.57	16.96	56.25
*Chimonanthus zhejiangensis*	63.27	17.85	53.13
*Chimonanthus praecox*	55.55	16.96	41.96

### Phylogenetic Inference and Estimation of Divergence Times

**FIGURE 4:**
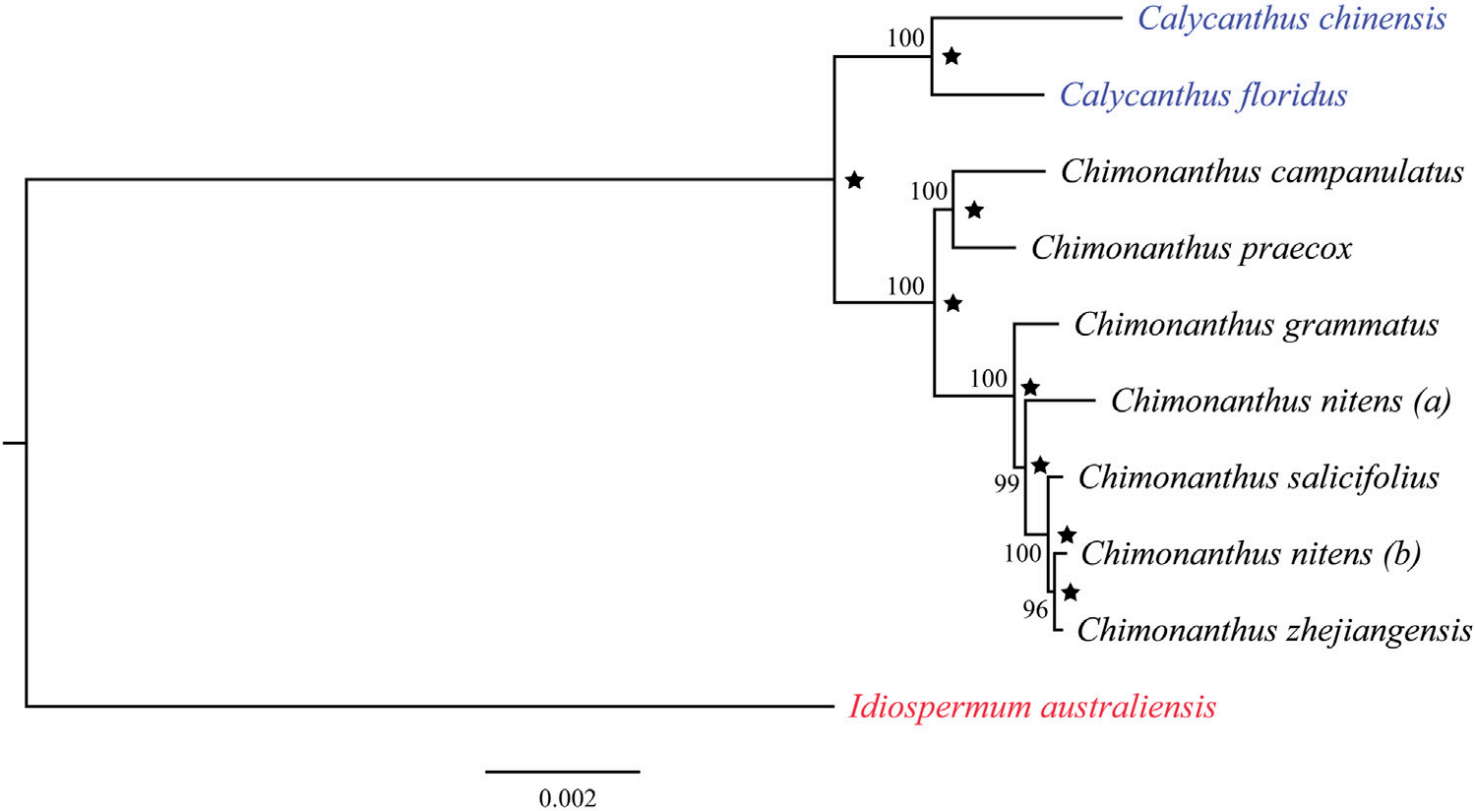
Phylogenetic relationships among nine species in the family Calycanthaceae based on the maximum-likelihood analyses and Bayesian inference. *Idiospermum australiense* was used as the outgroup. Numbers at each node are bootstrap support values in 100 replicates. Star indicates Bayesian posterior probabilities above 0.95 from the BEAST analyses.

A maximum likelihood tree was reconstructed using 10 chloroplast genomes in the family Calycanthaceae from all three genera. These included all six recognized species of *Chimonanthus*, two of the three *Calycanthus* species, and the only *Idiospermum* species. The *C. nitens* chloroplast genome was represented twice in this analysis: “*a*” was taken from NCBI (Dong et al., 2020), and “*b*” was assembled in this study (LIU2047, Supplementary Table S1). The multiple sequence alignment excluded one IR copy (IRb) and indels in the alignment, and was 127,010 nucleotides long, wherein 123,302 nucleotides (97.1%) were invariant sites. Best fit model on the data was found to be TVM + I + G4. The resulting phylogeny shows the monophyly of *Chimonanthus* that is sister to *Calycanthus*. However, *Chimonanthus nitens* is paraphyletic with accession *(b)* forming a clade with *C. zhejiangensis* but distinct from *C. nitens* *(a)* (Figure 4). The Bayesian inference (BI) using BEAST (Drummond and Rambaut, 2007) generated the same topology, and Figure 4 also shows the posterior probabilities of each node/clade.

**FIGURE 5:**
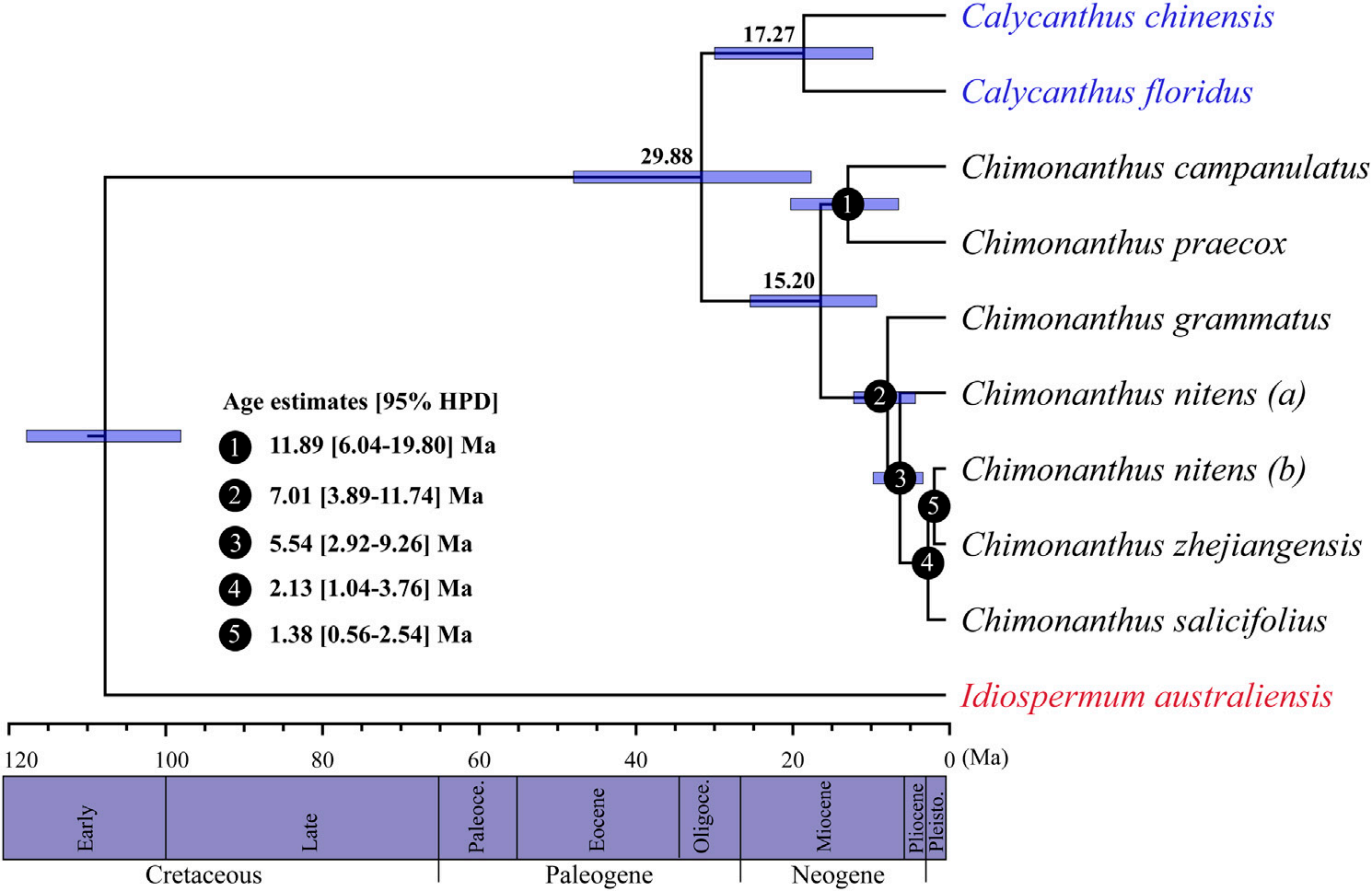
BEAST-derived chronograms of Calycanthaceae based on whole chloroplast genome sequences. Blue bars indicate the 95% highest posterior density (HPD) credibility intervals for node ages (Ma). Estimated divergence times of *Chimonanthus* species are indicated at the nodes (nodes 1–5).

Based on whole cp genome sequences, the BEAST analyses (Drummond and Rambaut, 2007) estimated the divergence between *Chimonanthus* and *Calycanthus* to be 29.88 Ma (95% HPD: 17.19–45.50), and that between *Calycanthus chinensis* and *Calycanthus floridus* as 17.27 Ma (95% HPD: 9.31–29.50; Figure 5). Within *Chimonanthus*, the divergence between the *C. praecox-campanulatus* clade and the clade of the rest of the four species of *Chimonanthus* was 15.20 Ma (95% HPD: 8.82–24.96), that between *C. praecox* and *C. campanulatus* was 11.89 Ma (node 1; 95% HPD: 6.04–19.80). The split of *C. grammatus* and the clade of *C. nitens*, *C. zhejiangensis*, and *C. salicifolius* occurred in the late Miocene, with an estimated age of 7.01 Ma (node 2; 95% HPD: 3.89–11.74). *C. nitens* *(a)* diverged from *C. salicifolius*, *C. zhejiangensis*, and *C. nitens* *(b)* at 5.54 Ma (node 3; 95% HPD: 2.92–9.26). The divergence time of *C. salicifolius* and that of *C. zhejiangensis* and *C. nitens* *(b)* occurred in the Pleistocene, with an estimated age of 2.13 Ma (node 4; 95% HPD: 1.04–3.76) and 1.38 Ma (node 5; 95% HPD: 0.56–2.54), respectively (Figure 5) [Supplementary Table S8 (A)]. The estimates for the divergence times of the different nodes through fossil record calibration showed slightly more recent estimates for each node [Supplementary Table S8 (B)].

## Discussion

In this study, we have assembled chloroplast genomes for five *Chimonanthus* species, and compared them with the two previously reported species of the genus (Dong et al., 2020). The chloroplast genomes of *Chimonanthus* species range in size from 153,010 to 153,299 bp with no obvious structural variation among the taxa. The organization and gene content of chloroplast genomes are similar among the studied species, consistent with previously reported genomes of Calycanthaceae (Goremykin et al., 2003; Dong et al., 2020). We have also evaluated correlations among mutational events, performed phylogenetic analyses in Calycanthaceae based on whole cp genomes, and explored their divergence history. These comparative chloroplast genomic analyses are important to provide insights into the genome organization and evolutionary history of species within the genus *Chimonanthus* and family Calycanthaceae.

### Chloroplast Genome Evolution of *Chimonanthus*

The *infA* gene (translation initiation factor 1), which has been independently lost multiple times during land plant evolution (Millen et al., 2001), is present in all *Chimonanthus* species in this study. This gene was also found in one species of *Calycanthus* (Goremykin et al., 2003) suggesting that the presence of *infA* in the chloroplast genome might be an ancestral condition in the family Calycanthaceae.

Chloroplast genomes are conserved in most plant lineages, but the expansion and contraction of the border regions between SC and IR regions contribute to variation in chloroplast genome lengths (Li et al., 2013; Dong et al., 2016; Menezes et al., 2018; Henriquez et al., 2020a; Abdullah et al., 2020c). Higher variation at the boundaries of single copy and inverted repeats is found in comparisons of deeply diverged lineages; conversely, those species that evolved recently have less variation at the boundary regions (Abdullah S. et al., 2019; Iram et al., 2019; Henriquez et al., 2020b; Shahzadi et al., 2020). In the current study, *Chimonanthus* species showed resemblance of this variational pattern at the junctions of single copy and inverted repeats. This might be indicative of recent evolutionary divergences among *Chimonanthus* species.

Codon usage analysis is essential to understand genome structure, evolutionary processes, and selection pressure on the genes (Morton, 1998; Goodarzi et al., 2008). The degenerative property of the genetic code shows that more than one codon can encode a single amino acid. Codons that encode a single amino acid are known as synonymous codons. Codon usage analysis in *Chimonanthus* species showed a bias for A/T-ending codons at 3′ end. This phenomenon has been mostly observed in many sequenced chloroplast genomes of land plants and may be due to A/T-rich chloroplast genome content (Amiryousefi et al., 2018b; Abdullah I. et al., 2019, Abdullah et al., 2019 S.; Mehmood et al., 2020a, 2020b). The high similarity exists in the codon usage of closely related species or of the same lineage, which provides insight into the evolution of plants, and the species of the same genus have high similarity compared with family-level comparison (Abdullah et al., 2020e; Abdullah et al., 2021). Hence, the high similarity in codon usage of *Chimonanthus* species showed their close evolutionary relationships.

Simple sequence repeats (SSRs) in the chloroplast genome can serve as highly informative genetic markers and are therefore often used in analysis of genetic variation, taxonomy, parentage analysis, functional diversity, linkage and comparative mapping, and evolutionary studies in various plant species (McCouch et al., 2002; Shirasawa et al., 2013; Xu et al., 2013; Ren et al., 2014; George et al., 2015; Yang et al., 2016; Zhu et al., 2016). We observed an abundance of mononucleotide repeats with A/T motif and of dinucleotide repeats with AT/AT motif in the chloroplast genome of *Chimonanthus* species. A similar pattern of SSR distribution was also reported in chloroplast genomes of other plant lineages (Du et al., 2017; Huo et al., 2019). The presence of abundant SSR loci in the genome suggests the potential utility for future population genetic work.

The Ka/Ks is important in evolutionary studies, as it reveals the selection pressure on protein-coding genes (Nazareno et al., 2015). The Ka/Ks < 1 describes purifying selection, Ka/Ks = 1 reveals neutral selection, and Ka/Ks > 1 shows positive selection (Lawrie et al., 2013). Due to higher Ks than Ka, we observed Ka/Ks < 1 for most of the protein-coding genes in *Chimonanthus* species. These results are consistent with previous studies of angiosperm chloroplast genomes, as purifying selection pressure mostly acts on the genes of chloroplast genomes (Cheng et al., 2017; Abdullah et al., 2020e; Shahzadi et al., 2020). The genes that showed higher Ka/Ks value are *rpoB* and *atpF* in *C. campanulatus*. This positive selection might confer some selective advantage to *C. campanulatus*.

The weak to strong correlations among mutational events including substitutions, InDels, and oligonucleotide repeats have been reported in the family Araceae (Ahmed et al., 2012; Abdullah et al., 2020a), Malvaceae (Abdullah et al., 2020d), between two species of *Cephalotaxus* (Cephalotaxaceae) (Yi et al., 2013), and in various species of *Dendrobium* (Orchidaceae) (Li et al., 2020). The strong correlations were observed at the family and subfamily levels (Abdullah et al., 2020d; 2020a), whereas weak correlations were observed among closely related species such as in pairwise comparisons of *Symplocarpus* (Abdullah et al., 2020a), *Theobroma* (Abdullah et al., 2020d), and *Cephalotaxus* (Yi et al., 2013). In the current study, the phylogenetic analyses revealed very close relationships among the species within *Calycanthus* and *Chimonanthus*. Therefore, we were interested in seeing how the mutational events are correlated. Our study again confirms that weak correlations exist among the closely related species. Previously, it was suggested that existence of fewer mutations between the compared species and incomplete lineage sorting may lead to low correlation coefficient (Abdullah et al., 2020a). Hence, the low correlations can be expected in the closely related species with very short divergence events due to availability of less time to InDels and repeats to generate mutations, as the mutations arise by errors in replications due to the presence of InDels or existence of a high number of repeats in regions of the genome (Tian et al., 2008; McDonald et al., 2011).

### Phylogenetic Relationships

**FIGURE 6:**
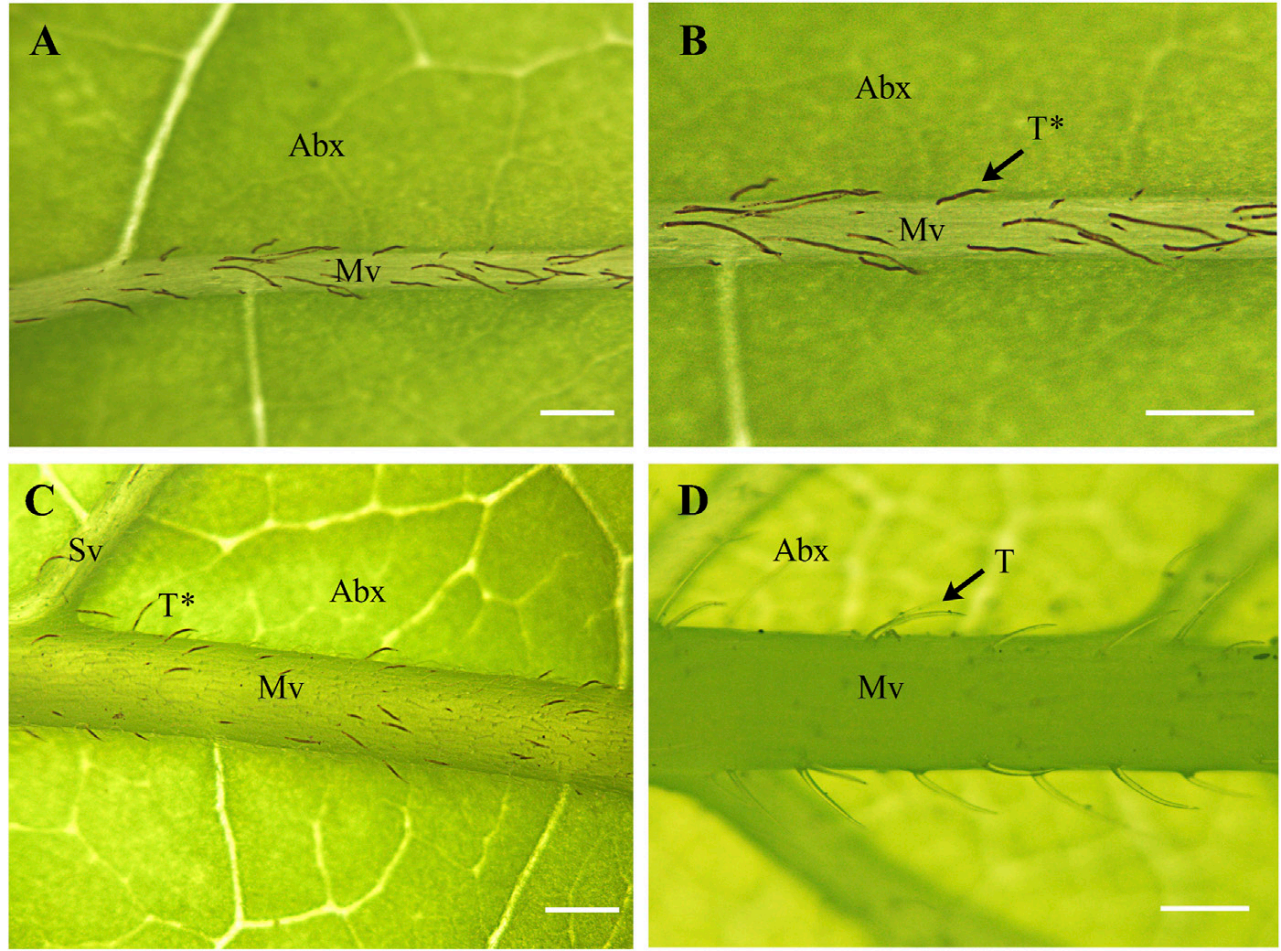
Structure and distribution of unicellular trichomes in Calycanthaceae. **(A,B)** *Calycanthus chinenses* **(C)** *Calycanthus floridus*; and **(D)** *Chimonanthus praecox*. Abx, abaxial; Mv, midvein; Sv, secondary vein; T, transparent trichomes; T*, dark brownish (non-transparent) trichomes. Scale bars **(A–D)** = 200 µm. Abaxial leaf surfaces were examined using a Nikon SMZ800 stereomicroscope.

Our phylogenetic analyses of Calycanthaceae (Figure 4) support the monophyly of both *Calycanthus* and *Chimonanthus*. *Calycanthus floridus* is sister to *Calycanthus chinensis*; however, the inclusion of *Calycanthus occidentalis* is needed in future studies to test the relationships within *Calycanthus*. Although *Calycanthus chinensis* endemic to Zhejiang, China, is considered morphologically highly distinct from the two North American species (Cheng and Chang, 1964), we have detected that it shares unusually similar dark brownish color trichomes with *Calycanthus floridus*, on the abaxial surface of the leaf midvein. On the other hand, species in *Chimonanthus*, e.g., *C. praecox* (other species not shown), share transparent trichomes distributed on the midvein of the abaxial leaf surface (Figure 6). The dark brownish color of trichomes may be due to the presence of biominerals (Ensikat et al., 2017), which may also provide phylogenetic signals (Mustafa et al., 2018). However, the confirmation and detailed patterns of biomineralization particularly in *Calycanthus* will require further investigation.

Within *Chimonanthus*, *C. praecox* and *C. campanulatus* form a clade, consistent with previous studies (Zhou et al., 2006, Zhou et al., 2007). The *Chimonanthus praecox*–*C. campanulatus* clade is sister to the clade of the remaining four species of the genus. *Chimonanthus grammatus* is sister to the clade of *C. salicifolius*, *C. zhejiangensis*, and *C. nitens*. *Chimonanthus nitens* is paraphyletic and it is closely related to *C. salicifolius* and *C. zhejiangensis*. In the previous study by Zhou et al. (2006), the relationships among the four species *C. salicifolius*, *C. grammatus*, *C. zhejiangensis*, *and C. nitens* were difficult to resolve. These species show high similarities in morphology and have been difficult to distinguish based on morphological characters (Chen, 1998; Dai et al., 2012). We sampled *C. nitens (b)* from the natural population located in Jiangxi, China (Supplementary Table S1), and it may represent an ecotype. Based on electrochemical fingerprint data, Xu et al. (2020) suggested that even *C. zhejiangensis* and *C. grammatus* may represent two ecotypes or varieties of *C. nitens*, and the small morphological variations between these three species may be due to environmental factors and may not be used for distinguishing the species. Chloroplast sequence data have also been used to suggest a particular species (*Colocasia formosana*) as an ecotype of another species (*Colocasia esculenta*) (Ahmed et al., 2020). However, confirmation of ecotypes requires reciprocal transplant experiments to link their establishment with local adaptation (Lowry, 2012). The complex species relationship in *Chimonanthus* and paraphyly of *C. nitens* in particular may have also arisen because of interspecific hybridization and introgression (Rieseberg and Brouillet, 1994; Hegarty and Hiscock, 2005; Mallet, 2005; Vriesendorp and Bakker, 2005). The reticulate phylogenetic relationships of closely related species caused by these naturally occurring processes present challenges to species delimitation (Mallet et al., 2016; Pennisi, 2016). Another cause of complex phylogenetic relationships within a group is incomplete lineage sorting, which cannot be ruled out in our case. Both incomplete lineage sorting and interspecific hybridization/introgression may have resulted in shared genetic variation (De Queiroz, 2007; Zhou et al., 2017). Both phenomena are observed frequently in taxa that are products of incipient radiation (Gavrilets and Losos, 2009; Goetze et al., 2017). Further studies at the population and species levels using both nuclear and chloroplast markers are required to test the alternate hypotheses of hybridization among distinct species and incomplete lineage sorting due to incipient radiation of *C. nitens* and its close relatives, eventually leading to the well-tested species delimitations in *Chimonanthus*.

### Diversification History of *Chimonanthus* in Eastern Asia

Overall, the species of *Chimonanthus* (except *Chimonanthus praecox*) are restricted to the subtropical regions of China, and our analyses indicate that early diversifications of most extant *Chimonanthus* species might have occurred during the middle and late Miocene (Figure 5, node 1, node 2, and node 3). This period corresponds to the intensification of the Eastern Asian monsoon and climate changes (Zhisheng et al., 2001; Sun and Wang, 2005), which might have contributed to the rapid radiation of many Eastern Asian plants, including subtropical lineages. For example, the initial diversification of subtropical *Cyclocarya paliurus* was associated with the intensification of Eastern Asian monsoon in the middle Miocene, which provided suitable climatic conditions that facilitated its survival in Southwestern China (Kou et al., 2016). Similarly, climatic fluctuations during late Miocene might have triggered the rapid radiation of *Quercus arbutifolia* (Fagaceae) inhabiting subtropical montane cloud forests (MCFs) in Southern China (Xu et al., 2016). Speciation event in the genus *Cercidiphyllum* at the Miocene/Pliocene boundary (Qi et al., 2012) and divergence of *Tetracentron sinense* by late Miocene global cooling (Sun et al., 2014) also suggest that pre-Quaternary climate changes might have contributed to the diversification of temperate and subtropical plants in Eastern Asia.

Zhou et al. (2006) reported that several species within *Chimonanthus* diverged recently approximately 1–2 Ma ago. Here we used whole cp genomes sequences to enhance the phylogenetic resolution and estimate the divergence times of *Chimonanthus* species, with all species sampled. The three species, *C. salicifolius*, *C. nitens* *(b)*, and *C. zhejiangensis* were found to have diverged relatively recently, with *C. salicifolius* diverging from the other two roughly 2.13–1.88 Ma (node 4 and node 5 in Figure 5), in the Pleistocene, which also agrees with the estimates of Zhou et al. (2006). Our results, hence, support active recent speciation events of an ancient lineage in the subtropical forest biome in Eastern China. The subtropical forests in Eastern China have been well known as an important refugia for the Eastern Asian flora (Wu, 1980) and for many Eastern Asian–North American disjunct lineages (Wen, 1999). Our results strongly showcase the importance of the subtropical forests as recent diversification centers for endemic elements of the Eastern Asian flora (see also Qiu et al., 2011; Wang H. X. et al., 2020).

## Data Availability

The datasets presented in this study can be found in online repositories. The names of the repository/repositories and accession number(s) can be found in the article. Moreover, additional data generated and analyzed are provided as Supplementary Material.

## Supplementary Material

Supplementary Figure S1 Distribution of repeats in the six *Chimonanthus* chloroplast genomes. **(A)** Represents distribution of microsatellite repeats in different regions of the chloroplast genomes. **(B)** Reputer results showing the number of forward, reverse, complimentary and palindromic oligonucleotide repeats in *Chimonanthus* chloroplast genomes.

Supplementary Table 1 Collection location and voucher specimen number of the studied *Chimonanthus* species.

Supplementary Table 2 Summary statistics of the data. Average coverage depth obtained after mapping the short reads to their respective assembled chloroplast genomes using BWA and visualizing in Tablet.

Supplementary Table 3 List of genes found in the chloroplast genomes of *Chimonanthus* species.

Supplementary Table 4 Frequencies and percentages of nucleotides and amino acids, and codon usage for the six Chimonanthus chloroplast genomes.

Supplementary Table 5 Distribution of microsatellite repeats in the six *Chimonanthus* chloroplast genomes.

Supplementary Table 6 Non-synonymous (Ka) versus synonymous (Ks) substitutions of 78 protein coding genes. Cc: *C. campanulatus*; Cg: *C. grammatus*; Cn: *C. nitens* (b); Cs: *C. salicifolius*; Cz: *C. zhejiangensis*.

Supplementary Table 7 Distributions of substitutions, InDels, and oligonucleotide repeats in bins of 250 bp in pairwise comparisons at family and genus level.

Supplementary Table 8 Divergence time estimates (in million years) for Calycanthaceae with the highest posterior density (HPD) interval limits. (A) The minimum age of Calycanthaceae was set to minimally 108 Mya (secondary calibration). (B) The minimum age of Calycanthaceae was set to minimally 90 Mya (fossil record).
