# Supplementary material for: Comparative Chloroplast Genome Analyses of the Winter-Blooming Eastern Asian Endemic Genus Chimonanthus (Calycanthaceae) With Implications For Its Phylogeny and Diversification
Source: Front Genet. 2021 Nov 30;12:709996. doi: 10.3389/fgene.2021.709996 (PMC8670589; doi:10.3389/fgene.2021.709996)
Supplement: Supplementary file 1 [file Table1.docx]

**Supplementary Table S1**: Collection location and voucher specimen number of the studied *Chimonanthus* species.

| Species | Voucher No. | Latitude | Longitude | City | Province | Country |
| --- | --- | --- | --- | --- | --- | --- |
| *Chimonanthus grammatus* | LIU2017 | 25.283328 | 115.457356 | Anyuan | Jiangxi | China |
| *Chimonanthus zhejiangensis* | LIU2141 | 28.139538 | 119.037272 | Longquan | Zhejiang | China |
| *Chimonanthus salicifolius* | LIU2112 | 28.775869 | 118.129215 | Yushan | Jiangxi | China |
| *Chimonanthus campanulatus* | CH-CAM | 30.477527 | 114.366694 | Wuhan | Hubei | China |
| *Chimonanthus nitens* | LIU2047 | 27.903552 | 117.202939 | Guixi, Lengshui | Jiangxi | China |
