# Supplementary material for: Comparative Chloroplast Genome Analyses of the Winter-Blooming Eastern Asian Endemic Genus Chimonanthus (Calycanthaceae) With Implications For Its Phylogeny and Diversification
Source: Front Genet. 2021 Nov 30;12:709996. doi: 10.3389/fgene.2021.709996 (PMC8670589; doi:10.3389/fgene.2021.709996)
Supplement: Supplementary file 2 [file Table8.docx]

**Supplementary Table S8:** Divergence time estimates (in million years) for Calycanthaceae with the highest posterior density (HPD) interval limits. (A) The minimum age of Calycanthaceae was set to minimally 108 Mya (secondary calibration). (B) The minimum age of Calycanthaceae was set to minimally 90 Mya (fossil record).

| **Table S8 (A)** |  |  |  |
| --- | --- | --- | --- |
| **Splits** | **95% HPD** | **Median** | **Time series (Median)** |
| *Calycanthus / Chimonanthus* | 17.19 – 45.50 | 29.88 | Oligocene |
| *Cal. chinensis / Cal. floridus* | 9.31 – 29.50 | 17.27 | middle / early Miocene |
| *C. praecox–C. campanulatus /* other *Chimonanthus* species | 8.82 – 24.96 | 15.20 | mid-Miocene |
| *C. campanulatus / C. praecox* | 6.04 – 19.80 | 11.89 | late Miocene |
| *C. grammatus / other Chimonanthus species* | 3.89 – 11.74 | 7.01 | late Miocene |
| *C. nitens (a) / other Chimonanthus species* | 2.92 – 9.26 | 5.54 | late Miocene |
| *C. salicifolius / C. zhejiangensis–C. nitens (b)* | 1.04 – 3.76 | 2.13 | Pleistocene |
| *C. zhejiangensis / C. nitens (b)* | 0.56 – 2.54 | 1.38 | Pleistocene |
| **Table S8 (B)** |  |  |  |
| **Splits** | **95% HPD** | **Median** | **Time series (Median)** |
| *Calycanthus / Chimonanthus* | 15.65 – 41.59 | 26.59 | Oligocene |
| *Cal. chinensis / Cal. floridus* | 8.22 – 25.83 | 15.35 | mid-Miocene |
| *C. praecox–C. campanulatus /* other *Chimonanthus* species | 8.11 – 21.75 | 13.49 | mid-Miocene |
| *C. campanulatus / C. praecox* | 5.11 – 17.09 | 10.58 | late Miocene |
| *C. grammatus / other Chimonanthus species* | 3.44 – 10.19 | 6.25 | late Miocene |
| *C. nitens (a) / other Chimonanthus species* | 2.69 – 8.05 | 4.94 | late Miocene / Pliocene |
| *C. salicifolius / C. zhejiangensis–C. nitens (b)* | 0.91 – 3.25 | 1.88 | Pleistocene |
| *C. zhejiangensis / C. nitens (b)* | 0.53 – 2.20 | 1.21 | Pleistocene |
