# Supplementary figures and images for: Comparative Chloroplast Genome Analyses of the Winter-Blooming Eastern Asian Endemic Genus Chimonanthus (Calycanthaceae) With Implications For Its Phylogeny and Diversification
Source: Front Genet. 2021 Nov 30;12:709996. doi: 10.3389/fgene.2021.709996 (PMC8670589; doi:10.3389/fgene.2021.709996)

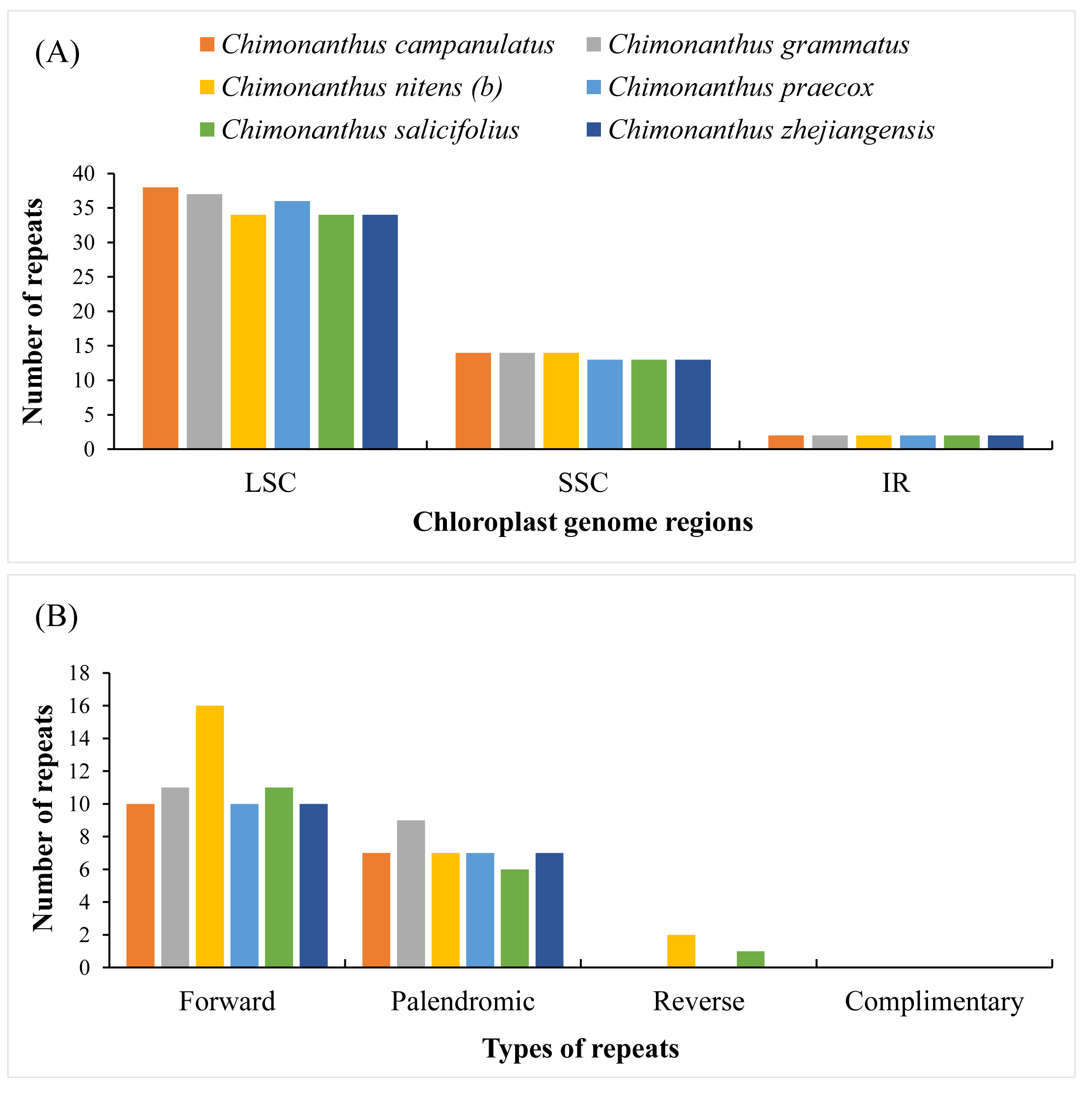

Supplement: Supplementary file 3 [file Image1.JPEG]
