# Supplementary material for: Comparative Chloroplast Genome Analyses of the Winter-Blooming Eastern Asian Endemic Genus Chimonanthus (Calycanthaceae) With Implications For Its Phylogeny and Diversification
Source: Front Genet. 2021 Nov 30;12:709996. doi: 10.3389/fgene.2021.709996 (PMC8670589; doi:10.3389/fgene.2021.709996)
Supplement: Supplementary file 5 [file Table2.docx]

**Supplementary Table S2**. Summary statistics of the data. Average coverage depth obtained after mapping the short reads to their respective assembled chloroplast genomes using BWA and visualizing in Tablet.

| Species | Data (Gb) x 2 (paired-end run) | Short reads (Millions) x 2 | Average coverage depth (x) | NCBI Accession number |
| --- | --- | --- | --- | --- |
| *C. campanulatus* | 9.5 | 25.87 | 6270 | MW166216 |
| *C. grammatus* | 8.7 | 23.7 | 2179 | MW166217 |
| *C. salicifolius* | 7.6 | 20.51 | 2494 | MW166218 |
| *C. zheijiangensis* | 7.2 | 19.58 | 1906 | MW166219 |
| *C. nitens (b)* | 7.1 | 19.21 | 1429 | MW166220 |
