# Supplementary material for: Comparative Chloroplast Genome Analyses of the Winter-Blooming Eastern Asian Endemic Genus Chimonanthus (Calycanthaceae) With Implications For Its Phylogeny and Diversification
Source: Front Genet. 2021 Nov 30;12:709996. doi: 10.3389/fgene.2021.709996 (PMC8670589; doi:10.3389/fgene.2021.709996)
Supplement: Supplementary file 6 [file Table5.docx]

**Supplementary Table S5:** Distribution of microsatellite repeats in the six *Chimonanthus* chloroplast genomes.

| **Types** | **Repeats** | ***C. cam*** | ***C. gra*** | ***C. nit (b)*** | ***C. pra*** | ***C. sal*** | ***C. zhe*** | **Average** |
| --- | --- | --- | --- | --- | --- | --- | --- | --- |
| Mono | A/T | 29 | 28 | 27 | 28 | 26 | 26 | 27.33 |
|  | C/G | 1 | 2 | 1 | 1 | 1 | 1 | 1.17 |
| Di | AC/GT | 1 | 1 | 1 | 1 | 1 | 1 | 1 |
|  | AG/CT | 2 | 1 | 2 | 2 | 2 | 2 | 1.83 |
|  | AT/AT | 5 | 5 | 5 | 5 | 5 | 5 | 5 |
| Tri | AAG/CTT | 1 | 1 |  | 1 |  |  | 0.5 |
|  | AAT/ATT | 3 | 2 | 2 | 3 | 2 | 2 | 2.33 |
| Tetra | AAAC/GTTT | 1 | 1 | 1 | 1 | 1 | 1 | 1 |
|  | AAAG/CTTT | 1 | 1 | 1 | 1 | 1 | 1 | 1 |
|  | AAAT/ATTT | 1 | 1 | 1 | 1 | 1 | 1 | 1 |
|  | AAGG/CCTT | 1 | 1 | 1 | 1 | 1 | 1 | 1 |
|  | AATC/ATTG | 1 | 1 | 1 | 1 | 1 | 1 | 1 |
|  | AATG/ATTC | 1 | 1 | 1 | 1 | 1 | 1 | 1 |
|  | AGAT/ATCT | 2 | 2 | 2 | 2 | 2 | 2 | 2 |
| Penta | AAACT/AGTTT | 1 | 1 | 1 | 1 | 1 | 1 | 1 |
|  | AAATC/ATTTG | 1 | 1 | 1 |  | 1 | 1 | 0.83 |
|  | AACTT/AAGTT | 1 |  |  |  |  |  | 0.17 |
|  | AATAG/ATTCT | 1 | 1 | 1 | 1 | 1 | 1 | 1 |
| Hexa | AAAAAG/CTTTTT |  | 1 |  |  |  |  | 0.17 |
|  | AATACT/AGTATT |  | 1 | 1 |  | 1 | 1 | 0.67 |
| Total |  | 54 | 53 | 50 | 51 | 49 | 49 |  |
