# Supplementary material for: Comparative Chloroplast Genome Analyses of the Winter-Blooming Eastern Asian Endemic Genus Chimonanthus (Calycanthaceae) With Implications For Its Phylogeny and Diversification
Source: Front Genet. 2021 Nov 30;12:709996. doi: 10.3389/fgene.2021.709996 (PMC8670589; doi:10.3389/fgene.2021.709996)
Supplement: Supplementary file 8 [file Table3.docx]

**Supplementary Table S3**: List of genes found in the chloroplast genomes of *Chimonanthus* species.

| Category | Gene group | Name of genes | |  |  |  |
| --- | --- | --- | --- | --- | --- | --- |
| Self-replication (60) | Ribosomal RNA genes | *rrn5* | *rrn4.5* | *rrn16* | *rrn23* |  |
|  | Transfer RNA genes | *trnA-*UGC* | *trnC-GCA* | *trnD-GUC* | *trnE-UUC* | *trnF-GAA* |
|  |  | *trnG-GCC** | *trnG-UCC* | *trnH-GUG* | *trnI-CAU* | *trnI-GAU** |
|  |  | *trnK-UUU** | *trnL-CAA* | *trnL-UAA** | *trnL-UAG* | *trnfM-CAU* |
|  |  | *trnM-CAU* | *trnN-GUU* | *trnP-UGG* | *trnQ-UUG* | *trnR-ACG* |
|  |  | *trnR-UCU* | *trnS-GCU* | *trnS-GGA* | *trnS-UGA* | *trnT-GGU* |
|  |  | *trnT-UGU* | *trnV-GAC* | *trnV-UAC** | *trnW-CCA* | *trnY-GUA* |
|  | Small subunit of ribosome | *rps2* | *rps3* | *rps4* | *rps7* | *rps8* |
|  |  | *rps11* | *rps12*** | *rps14* | *rps15* | *rps16** |
|  |  | *rps18* | *rps19* |  |  |  |
|  | Large subunit of ribosome | *rpl2** | *rpl14* | *rpl16** | *rpl20* | *rpl22* |
|  |  | *rpl23* | *rpl32* | *rpl33* | *rpl36* |  |
|  | RNA polymerase subunits | *rpoA* | *rpoB* | *rpoC1** | *rpoC2* |  |
|  | Translation initiation factor | *infA* |  |  |  |  |
| Genes for photosynthesis (46) | Large subunit of RuBisCO | *rbcL* |  |  |  |  |
|  | Subunits of photosystem I | *psaA* | *psaB* | *psaC* | *psaI* | *psaJ* |
|  | Subunits of photosystem II | *psbA* | *psbB* | *psbC* | *psbD* | *psbE* |
|  |  | *psbF* | *psbH* | *psbI* | *psbJ* | *psbK* |
|  |  | *psbL* | *psbM* | *psbN* | *psbT* | *lbhA* |
|  | Photosytem I assembly protien | *ycf4* |  |  |  |  |
|  | Subunits of ATP synthase | *atpA* | *atpB* | *atpE* | *atpF** | *atpH* |
|  |  | *atpI* |  |  |  |  |
|  | Cytochrome b/f compelx | *petA* | *petB** | *petD** | *petG* | *petL* |
|  |  | *petN* |  |  |  |  |
|  | C-type cytochrome synthesis | *ccsA* |  |  |  |  |
|  | NADH dehydrogenase subunits | *ndhA** | *ndhB** | *ndhC* | *ndhD* | *ndhE* |
|  |  | *ndhF* | *ndhG* | *ndhH* | *ndhI* | *ndhJ* |
|  |  | *ndhK* |  |  |  |  |
| Other genes (7) | Maturase | *matK* |  |  |  |  |
|  | Protease | *clpP*** |  |  |  |  |
|  | Envelope membrane protien | *cemA* |  |  |  |  |
|  | Subunit of acetyl-CoA | *accD* |  |  |  |  |
|  | Hypothetical open reading frames | *ycf1* | *ycf2* | *ycf3*** |  |  |

Note: *, Genes containing one intron; **, Genes containing two introns.
